# *CVRanalysis*: a free software for analyzing cardiac, vascular and respiratory interactions

**DOI:** 10.3389/fphys.2023.1224440

**Published:** 2024-01-04

**Authors:** Vincent Pichot, Christophe Corbier, Florian Chouchou, Jean-Claude Barthélémy, Frédéric Roche

**Affiliations:** ^1^ SAINBIOSE U1059, Inserm, Saint-Etienne Jean-Monnet University, Clinical Physiology and Exercise, CHU of Saint-Etienne, Saint-Etienne, France; ^2^ LASPI EA3059, Saint-Etienne Jean-Monnet University, Roanne Technology University Institute, Roanne, France; ^3^ IRISSE EA4075, UFR SHE, University of La Réunion, Le Tampon, France

**Keywords:** cardiovascular, cardiorespiratory, autonomic nervous system, baroreflex, heart rate variability, Granger causality

## Abstract

**Introduction:** Simultaneous beat-to-beat R-R intervals, blood pressure and respiration signals are routinely analyzed for the evaluation of autonomic cardiovascular and cardiorespiratory regulations for research or clinical purposes. The more recognized analyses are i) heart rate variability and cardiac coherence, which provides an evaluation of autonomic nervous system activity and more particularly parasympathetic and sympathetic autonomic arms; ii) blood pressure variability which is mainly linked to sympathetic modulation and myogenic vascular function; iii) baroreflex sensitivity; iv) time-frequency analyses to identify fast modifications of autonomic activity; and more recently, v) time and frequency domain Granger causality analyses were introduced for assessing bidirectional causal links between each considered signal, thus allowing the scrutiny of many physiological regulatory mechanisms.

**Methods:** These analyses are commonly applied in various populations and conditions, including mortality and morbidity predictions, cardiac and respiratory rehabilitation, training and overtraining, diabetes, autonomic status of newborns, anesthesia, or neurophysiological studies.

**Results:** We developed *CVRanalysis*, a free software to analyze cardiac, vascular and respiratory interactions, with a friendly graphical interface designed to meet laboratory requirements. The main strength of *CVRanalysis* resides in its wide scope of applications: recordings can arise from beat-to-beat preprocessed data (R-R, systolic, diastolic and mean blood pressure, respiration) or raw data (ECG, continuous blood pressure and respiratory waveforms). It has several tools for beat detection and correction, as well as setting of specific areas or events. In addition to the wide possibility of analyses cited above, the interface is also designed for easy study of large cohorts, including batch mode signal processing to avoid running repetitive operations. Results are displayed as figures or saved in text files that are easily employable in statistical softwares.

**Conclusion:** *CVRanalysis* is freely available at this website: anslabtools.univ-st-etienne.fr. It has been developed using MATLAB^®^ and works on Windows 64-bit operating systems. The software is a standalone application avoiding to have programming skills and to install MATLAB. The aims of this paper area are to describe the physiological, research and clinical contexts of *CVRanalysis*, to introduce the methodological approach of the different techniques used, and to show an overview of the software with the aid of screenshots.

## 1 Introduction

In the field of autonomic nervous system, ECG, blood pressure (BP) waveform and respiratory movements are simultaneously recorded routinely in humans for research or clinical purposes. From these recordings, beat-to-beat values of R-R intervals (RR) or heart rate (HR), systolic, mean and diastolic bood pressure (SBP, MBP and DBP, respectively), and respiration (RE) are extracted and used for the analyses of cardiovascular and cardiorespiratory regulations.

Usually these recordings are carried out over some minutes to several hours. After a basal measurement often made in the supine position (Radaelli et al., 1999) during which parasympathetic nervous system activity is predominant, a second test is generally realized to modify the sympathetic/parasympathetic autonomic nervous system equilibrium according to the studied physiological process. This task can be simply done by setting the subject in an upright position (Lucini et al., 2000; Monaghan et al., 2022), or performing handgrip exercise (Cui et al., 2021), cognitive stress test (Niizeki and Saitoh, 2012), pharmacological administration (mostly autonomic blockades) (Januel et al., 1995; Porta et al., 2013b), cold pressure test (Prakash et al., 2021), or lower body negative pressure (Butler et al., 1994), during which, respiration can be spontaneous or paced (Radaelli et al., 2004).

From these RR, BP and RE series, the most common calculated indices arise from heart rate variability (HRV) analysis which provides an estimation of autonomic nervous system activity, reactivity and equilibrium (Task Force of the European Society of Cardiology and the North American Society of Pacing and Electrophysiology, 1996; Rajendra Acharya et al., 2006). Time and frequency domain indices give estimations of parasympathetic and sympathetic activities and sympatho-vagal equilibrium (Akselrod et al., 1981; Pagani et al., 1984); and nonlinear methods such as fractal or entropy indices bring information on the complexity of autonomic regulations (Pincus and Goldberger, 1994; Peng et al., 1995; Voss et al., 1995; Porta et al., 1998; Richman and Moorman, 2000). In the same manner but less used than HRV, frequency domain analysis of beat-to-beat BP is mainly linked to sympathetic modulation and myogenic vascular function (Parati et al., 1990; Stauss, 2007).

Another popular and well used index is the cardiac baroreflex sensitivity (BRS) which represents the compensatory increase and decrease in RR due to spontaneous variation of BP (Kaufmann et al., 2020). The sequence and the frequency domain methods (Parlow et al., 1995; Pinna and Maestri, 2001; Pinna and Maestri, 2002; Reyes Del Paso et al., 2017) are historically the two reference methods; but some alternatives such as phase rectified signal averaging (Bauer et al., 2010) have proven their accuracy for baroreflex evaluation. These approaches are complemented by more recently developed methods. For non-steady state recordings, it is possible to analyze the transient evolution of autonomic activity and baroreflex using time-frequency methods such as wavelet transform (Pichot et al., 1999; Wiklund et al., 2002).

RR and BP variability as well as BRS indices are commonly applied to various fields such as morbidity and mortality risk stratification (La Rovere et al., 1998; Lucreziotti et al., 2000), aging (Milan-Mattos et al., 2018), sports fields to manage training (Pichot et al., 2005; Buchheit, 2014), overtraining (Mourot et al., 2004; Baumert et al., 2006) or cardiac rehabilitation (Malfatto et al., 1996; Badrov et al., 2019), metabolic syndrome (Assoumou et al., 2010; Endukuru et al., 2020), hypertension (Pikkujämsä et al., 1998; Del Colle et al., 2007), pain (Chouchou et al., 2011), diabetes dysautonomia evaluation (Petry et al., 2020) and autonomic maturation (Patural et al., 2019). Although, these indices provided new physiological insights and were strongly correlated to the incidence of fatal and nonfatal cardiovascular and all-cause events, a limitation of these methods resides in that they do not consider all closed loop interactions between RR, BP and RE involved during cardiovascular and cardiorespiratory regulations. Also, the spectral methods were subject to criticism, particularly concerning the estimation of sympathetic activity and sympatho-vagal balance (Billman, 2013). To override this limitation, causal mathematical analyses were introduced during the last decade to better understand the physiological relationships between RR, BP and RE signals (Schulz et al., 2013; Müller et al., 2016). The more documented and promising method is the Granger causality analysis, which provides indices of the causality links between each signal, thus allowing identification of supported specific physiological regulation mechanisms (Granger, 1969; Schulz et al., 2013; Müller et al., 2016). At the moment, these methods were validated in the laboratory (Porta et al., 2013b) and applied to several clinical fields such as anesthesia (Bassani et al., 2012; Porta et al., 2013a) or syncope (Faes et al., 2015; Schiatti et al., 2015), but major clinical studies using these new tools are still lacking.

Some softwares to analyze RR, BP and RE have been developed and improved during the last two decades. Usually, the applications are dedicated to the calculation of HRV or baroreflex indices. Some ready to use softwares are attached to a specific material distributed by a biomedical company and utilize their own file format and have to be paid, whereas others are free of charge (Kaufmann et al., 2011; Tarvainen et al., 2014; Pichot et al., 2016; Silva et al., 2020). Also, open source toolboxes for MATLAB, C or Python (Champeix, 2018; Mietus and Goldberger, 2018; Vollmer, 2020; Gomes, 2022) have given a large number of people access to methods for analyzing RR, BP and RE signals and thus improved knowledge in the field, but require programming skills. A recent article presented a detailed summary of the common softwares available for cardiovascular signal analyses (Silva et al., 2020).

In this article, we introduce *CVRanalysis*, a new free software available for research purposes that gathers the main recognized methods to analyze RR, BP and RE signals recorded simultaneously. The available analysis methods are HR and BP variability, BRS, wavelets analysis, classical and extended Granger causalities in time and frequency domains, and analyses on surrounding events. *CVRanalysis* software has been developed in the same manner as *HRVanalysis* (Pichot et al., 2016) and users will find similar menus and tools adapted for laboratory requirements such as various data importation formats, beat detection and correction, setting of specific areas or events, and batch analyses possibility. To our knowledge, there is no other free software gathering all these methods and proposing such laboratory tools, especially for RR, BP and RE signals analysis.

## 2 Methods to explore cardiac, vascular, and respiratory interactions

In this section, the reader will find a description of the methods used by the software. Of course, not all methods are present, as there is a wide choice in the literature but we believe we have included a selection of the most useful and widely used in the field. We have confined ourselves to describing the principles of the methods without detailing the algorithms and have only introduced the most commonly used physiological interpretations. All these methods have been validated and widely used in many fields, and the reader may wish to refer to the many articles that have been included in the text.

Please, refer to Tables 1–4 for a list of all indices calculated from each method and their abbreviations.

**TABLE 1:** Definition of abbreviations for calculated indices in *CVRanalysis* program.

Index	
Time domain	
NN20	Number of pairs of successive normal RR intervals that differ by more than 20 ms
pNN20	Proportion of NN20 divided by the total number of normal RR intervals
NN30	Number of pairs of successive normal RR intervals that differ by more than 30 ms
pNN30	Proportion of NN30 divided by the total number of normal RR intervals
NN50	Number of pairs of successive normal RR intervals that differ by more than 50 ms
pNN50	Proportion of NN50 divided by the total number of normal RR intervals
SDNN	Standard deviation of the normal RR (or BP) intervals
rMSSD	Root mean square of the successive RR (or BP) interval differences
Geometrical	
Triangular Index	Integral of the density of the RR (or BP) interval histogram divided by its height (Y)
TINN	Baseline width of the RR (or BP) interval histogram
X	Duration of the RR (or BP) at the peak of the density distribution histogram
Y	Height of the peak of the density distribution histogram
M	Right limit of the density distribution histogram
N	Left limit of the density distribution histogram
Frequency domain	
Ptot	Total power of the RR (or BP) spectrum
VLF	Absolute power of the very-low-frequency band
LF	Absolute power of the low-frequency band
HF	Absolute power of the high-frequency band
LFnu	Relative power of the low-frequency band
HFnu	Relative power of the high-frequency band
Empirical mode decomposition	
pLF1	Power associated with the mode closest to 0.1 Hz
pLF2	Power associated with the first mode with freq < LF1
pHF1	Power associated with the first mode with freq > LF1
pHF2	Power associated with the second mode with freq > LF1
IMAI1	Ratio between pLF1 and power of modes with freq > LF1
IMAI2	Ratio between pLF2 and power of modes with freq > LF1
Poincaré plot	
Centroïd	RR (or BP) value a the centre of the elipse
SD1	Poincaré plot standard deviation perpendicular to the line of identity
SD2	Poincaré plot standard deviation along the line of identity
SD1nu	Ratio between SD1 and centroid in %
SD2nu	Ratio between SD2 and centroid in %
Fractal	
α1_DFA_	Detrended fluctuation analysis which describes short-term fluctuations
α2_DFA_	Detrended fluctuation analysis which describes long-term fluctuations
H_DFA_	Slope of the DFA curve plotted in log-log
H_Higuchi_	Fractal dimension using Higuchi algorithm
H_Katz_	Fractal dimension using Katz algorithm
Hurst	Hurst exponent
Symbolic dynamic	
0V	Number of pattern showing 0 variations in the signal
0V%	Percentage of pattern showing 0 variations in the signal
1V	Number of pattern showing two successive equal points
1V%	Percentage of pattern showing two successive equal points
2LV	Number of pattern showing two variations in the same direction
2LV%	Percentage of pattern showing two variations in the same direction
2UV	Number of pattern showing two variations in opposite directions
2UV%	Percentage of pattern showing two variations in opposite directions
MP	Number of missing pattern
MP%	Percentage of missing pattern
Cardiac coherence	
HR coherence ratio	Heart rhythm coherence ratio
F(peak) Hz	Frequency of the maximum peak between 0.04 and 0.26 Hz expressed in Hz
F(peak) cycle/min	Frequency of the maximum peak expressed in cycle/s
F(peak) s	Frequency of the maximum peak expressed in s

**TABLE 2:** HRV, BPV and cardiac coherence indices calculated in *CVRanalysis* program.

RR series		SBP, MBP, DBP	
Index	Unit	Index	Unit
Time domain Eckberg (1983), Fouad et al. (1984), Cook et al. (1991), Kleiger et al. (1995), Task Force of the European Society of Cardiology and the North American Society of Pacing and Electrophysiology (1996), Rajendra Acharya et al. (2006)			
Mean RR	ms	Mean BP	mmHg
Mean HR	bpm		
NN20	n		
pNN20	%		
NN30	n		
pNN30	%		
NN50	n		
pNN50	%		
SDNN	ms	SD	mmHg
rMSSD	ms	rMSSD	mmHg
Geometrical Malik et al. (1989), Malik (1995), Task Force of the European Society of Cardiology and the North American Society of Pacing and Electrophysiology, (1996)			
Triangular Index	—	Triangular Index	—
TINN	ms	TINN	mmHg
X	ms	X	mmHg
Y	n	Y	mmHg
M	ms	M	mmHg
N	ms	N	mmHg
Frequency domain Akselrod et al. (1981), Pagani et al. (1984), Akselrod et al. (1985), Pomeranz et al. (1985), Pagani et al. (1986), Saul et al. (1990), Bigger et al. (1992), Triedman and Saul, (1994), Malliani et al. (1995), Keselbrener et al. (1996), Task Force of the European Society of Cardiology and the North American Society of Pacing and Electrophysiology, (1996), Eckberg (1997), Cooley et al. (1998), Pichot et al. (1999), Wiklund et al. (2002), Belova et al. (2007), Billman (2013), Billman et al. (2015)			
Ptot*	ms^2^	Ptot*	mmHg^2^
VLF*	ms^2^	VLF*	mmHg^2^
LF*	ms^2^	LF*	mmHg^2^
HF*	ms^2^	HF*	mmHg^2^
LF/HF*	—	LF/HF*	—
LFnu*	%	LFnu*	%
HFnu*	%	HFnu*	%
Empirical mode decomposition Balocchi et al. (2004)			
pLF1	ms^2^	pLF1	mmHg^2^
pLF2	ms^2^	pLF2	mmHg^2^
pHF1	ms^2^	pHF1	mmHg^2^
pHF2	ms^2^	pHF2	mmHg^2^
IMAI1	—	IMAI1	—
IMAI2	—	IMAI2	—
Poincaré plot Kamen et al. (1996), Balocchi et al. (2006)			
Centroïd	ms	Centroïd	mmHg
SD1	ms	SD1	mmHg
SD2	ms	SD2	mmHg
SD1/SD2	—	SD1/SD2	—
SD1nu	%	SD1nu	%
SD2nu	%	SD2nu	%
Fractal Katz (1988), Peng et al. (1995), Huikuri et al. (2000), Rajendra Acharya et al. (2006), Maestri et al. (2007b)			
α1_DFA_	—	α1_DFA_	—
α2_DFA_	—	α2_DFA_	—
H_DFA_	—	H_DFA_	—
H_Higuchi_	—	H_Higuchi_	—
H_Katz_	—	H_Katz_	—
Hurst	—	Hurst	—
Moments Yilmaz and Yildiz, (2010), Wolf et al. (1985)			
Skewness	—	Skewness	—
Kurtosis	—	Kurtosis	—
Largest Lyapunov exponent	—	Largest Lyapunov exponent	—
Entropy Pincus (1991), Porta et al. (1998), Richman and Moorman, (2000), Porta et al. (2001), Ferrario et al. (2004), Maestri et al. (2007a), Porta et al. (2007)			
Approximate entropy (AppEn)	—	Approximate entropy	—
Sample entropy (SampEn)	—	Sample entropy	—
Shanon Entropy (SE)	—	Shanon Entropy (SE)	—
Conditional Entropy (CE)	—	Conditional Entropy (CE)	—
Corrected CE (CCE)	—	Corrected CE (CCE)	—
Normalized CCE (NCCE)	—	Normalized CCE (NCCE)	—
ρ	—	ρ	—
Lempel-Ziv complexity	—	Lempel-Ziv complexity	—
Symbolic dynamic Porta et al. (2001), Guzzetti et al. (2005), Porta et al. (2007), Dantas et al. (2015)			
0V	—	0V	—
0V%	%	0V%	%
1V	—	1V	—
1V%	%	1V%	%
2LV	—	2LV	—
2LV%	%	2LV%	%
2UV	—	2UV	—
2UV%	%	2UV%	%
MP	—	MP	—
MP%	%	MP%	%
Cardiac coherence McCraty 2009, Russo et al. (2017)			
HR coherence ratio	—		
F(peak)	Hz		
F(peak)	cycle/min		
F(peak)	s		

*Indices also calculated in the time-frequency domain (wavelet analysis).

**TABLE 3:** Baroreflex indices calculated in *CVRanalysis* program.

Sequence method Parati et al. (1990), Parlow et al. (1995), Reyes Del Paso et al. (2017), Di Rienzo et al. (2001)	
BRS sensitivity (all)	ms/mmHg
Effectiveness (all)	%
Detected sequences (all)	n
Included sequences (all)	n
Excluded sequences (all)	n
BRS sensitivity (positive)	ms/mmHg
Effectiveness (positive)	%
Detected sequences (positive)	n
Included sequences (positive)	n
Excluded sequences (positive)	n
BRS sensitivity (negative)	ms/mmHg
Effectiveness (negative)	%
Detected sequences (negative)	n
Included sequences (negative)	n
Excluded sequences (negative)	n
Transfer function Javorka et al. (2021), Pinna and Maestri, (2001), Pinna 2002a, Pinna 2002b	
αLF	ms/mmHg
αHF	ms/mmHg
Phase LF	rad
Phase HF	rad
Delay LF	sec
Delay HF	sec
Phase rectified signal averaging Bauer et al. (2010), Muller et al. (2012)	
BRS PRSA original	ms
BRS PRSA normalized	ms/mmHg
Detected segments	n
SBP change	mmHg

*Indices also calculated in the time-frequency domain (wavelet analysis).

**TABLE 4:** Granger causality indices calculated in *CVRanalysis* program.

Model			
Fit VAR (RR)	%		
Fit VAR (BP)	%		
Fit VAR (RE)	%		
Lag model	n		
**Time domain causality (classic)**		**Time domain causality (extended)**	
Wiener, (1956), Granger (1969), Porta et al. (2013b), Faes (2014), Schiatti et al. (2015)			
BP→RR	[0–1]	BP→RR	[0–1]
BP←RR	[0–1]	BP←RR	[0–1]
BP↔RR	[0–1]	B0 (BP→RR)	—
BP ≠ RR	[0–1]	B0 (BP←RR)	—
BP→RE	[0–1]	BP→RE	[0–1]
BP←RE	[0–1]	BP←RE	[0–1]
BP↔RE	[0–1]	B0 (BP→RE)	—
BP ≠ RE	[0–1]	B0 (BP←RE)	—
RR→RE	[0–1]	RR→RE	[0–1]
RR←RE	[0–1]	RR←RE	[0–1]
RR↔RE	[0–1]	B0 (RR→RE)	—
RR ≠ RE	[0–1]	B0 (RR←RE)	—
Frequency domain causality (classic and extended) Kamiński and Blinowska, (1991), Baccala et al. (1998), Baccala and Sameshima, (2001), Faes and Nollo, (2010); Faes and Nollo, (2011), Faes et al. (2013a)			
BP→RR (HF)	[0–1]		
BP←RR (HF)	[0–1]		
BP→RR (LF)	[0–1]		
BP←RR (LF)	[0–1]		
BP→RE (HF)	[0–1]		
BP←RE (HF)	[0–1]		
BP→RE (LF)	[0–1]		
BP←RE (LF)	[0–1]		
RR→RE (HF)	[0–1]		
RR←RE (HF)	[0–1]		
RR→RE (LF)	[0–1]		
RR←RE (LF)	[0–1]		

### 2.1 Heart rate and blood pressure variability

#### 2.1.1 Heart rate variability

HRV is a well recognized method to explore autonomic nervous system activity for laboratory and clinical uses (Task Force of the European Society of Cardiology and the North American Society of Pacing and Electrophysiology, 1996; Rajendra Acharya et al., 2006; Billman, 2011; Billman et al., 2015). Indeed, except for nonsinus rhythms induced by atrial fibrillation or extrasystoles, the main variations in HR are due to the permanent beat-to-beat adaptations of parasympathetic and sympathetic activities on the heart (Hon and Lee, 1963; Sayers, 1973; Billman, 2011). The quantification of these variations then allows to evaluate autonomic activity (Akselrod et al., 1981). This is achieved by various methods: time and frequency domain, nonlinear, and time-frequency (Rajendra Acharya et al., 2006; Pichot et al., 2016). Each calculated index provides different information, but some are redundant (Kautzner and Hnatkova, 1995). Globally, the rapid variations concerning several beats are linked to parasympathetic activity (Akselrod et al., 1981; Pomeranz et al., 1985); slower variations with a period 7–25 s are due to both sympathetic and parasympathetic activities (Saul et al., 1990; Pagani et al., 1997); and very slow variations with a period 25–300 s are usually assimilated to parasympathetic activity, renin angiotensine system and thermoregulation (Taylor et al., 1998). Ultraslow variations have been shown but required 24-h recordings to be calculated (Bigger et al., 1992). Then, for example, from the time and frequency domain methods, both SDNN and Ptot give information on the total beat-to-beat variations, *i.e.,* an approximation of the global regulatory capacities of the autonomic nervous system; rMSSD, pNN50 and HF are linked to short-term beat-to-beat variations and are related to parasympathetic activity; the LF/HF ratio can be considered as an approximation of the sympatho-vagal balance (Pagani et al., 1984), although this index should be considered with caution (Billman, 2013).

Precautions must also be taken with regard to respiratory rate. Indeed, the physiological interpretations of the frequency domain indices described above are only valid if the subject’s respiratory frequency is in the high-frequency bandwidth, i.e., between 9 and 24 cycles per minute for HF bandwidth set between 0.15 and 0.40 Hz. For slower breathing rates, the peak of the LF will be contaminated by the effects of breathing and the HF will be artificially reduced. For respiration that is too fast, its effects will not be taken into account in the HF. It is also important to take into account the state in which the subject is: in the upright position or during hand-grip exercise, for example, the LF will be more influenced by sympathetic activity than in the supine position at rest, where parasympathetic activity will predominate.

A method that is often used to complement time domain analysis, the Poincaré plot is a graphical representation of the RR(n) as a function of RR(n + 1), modelled in the form of an elipse (Kamen et al., 1996). Then, the standard deviation of the RRs along the x = y-axis (index SD1) represents short-term variations in RR variability, while the standard deviation on the perpendicular axis (index SD2) represents longer term variability. To measure the relationship between these two indices, the value of SD1/SD2 is often used.

Also, unlike time and frequency domain methods, which identify known regulation mechanisms that respond to specific signal variations (the effect of breathing at high HF frequencies of RR and BP, or Meyer waves located at 0.1 Hz in the BP spectrum, for example,), nonlinear analyses permit to scrutinize the capacity of more complex regulatory mechanisms by the autonomic nervous system (Voss et al., 1995).

Among these, the analysis of fractality consists of quantifying the repetition of identical shapes but at different scales in the RR series (Peng et al., 1995; Bigger et al., 1996; Rajendra Acharya et al., 2006). Several approaches have been introduced to estimate the degree of fractality of HRV: the detrended fluctuation analysis (DFA) (Peng et al., 1995), the Hurst exponent, or the Higuchi (Higuchi, 1998) and Katz algorithms (Katz, 1988).

Entropy measures the complexity and regularity of patterns of different durations in the signal. Thus entropy will increase when a wide variety of patterns are identically distributed and decreases when these shapes are always the same. A number of algorithms have been developed to calculate these entropy indices: sample entropy, approximate entropy, Shannon entropy and the indices derived from it, conditional entropy, corrected conditional entropy, normalized corrected conditional entropy (Porta et al., 1998; Ferrario et al., 2004).

The symbolic dynamics allow to study short RR or BP variability pattern behavior (Porta et al., 2001). The principle of this method is to divide the signal into parts containing a fixed number of points (typically L = 3) and to digitize it (typically E = 6 levels). For each epoch obtained, the presence of predefined patterns is quantified (0V: no variations in the signal; 1V: two successive equal points; 2LV: two variations in the same direction; 2UV: two variations in opposite directions) from which their rates of occurrence in the complete signal are deduced.

Finally, we should also mention the largest Lyapunov exponent, which is used to measure how chaotic is the analyzed signal (Wolf et al., 1985). Its values will tend towards zero for physiological signals showing very small and slow variations, whereas they will increase when the variations are quickely larger and larger.

#### 2.1.2 Blood pressure variability

In clinical practice, BP variability usually represents from hour-to-hour to days, weeks, months and years variations (Nardin et al., 2019). In this context, an increase of variability is correlated with an increased risk of cardiovascular and all-cause mortality due to the alteration in cardiovascular regulatory mechanisms (Parati et al., 2013). However, very short-term, beat-to-beat BP variations have been analyzed and can be performed to assess cardiovascular regulation (Parati et al., 2020).

BP regulation is realized by different neurohormonal systems including the baroreflex system, the renin-angiotensin system, the shear stress-induced release nitric oxide from the endothelium, and the myogenic vascular response (Stauss, 2007). In addition to these regulatory mechanisms, independent and spontaneous variation of BP sympathetically mediated so called Mayer waves, which arise around 0.1 Hz, has also been demonstrated (Julien, 2020). These physiological systems induce variations in BP, which can be identified in a specific bandwidth of spectral analysis (Parati et al., 1990). In humans, myogenic vascular function affects both the very low frequencies (0.02–0.07 Hz) and the low frequencies (0.075–0.15 Hz); and sympathetic modulation can be seen only in the low frequencies and the LF/HF ratio (Stauss, 2007). The usual interpretation of high frequencies (0.15–0.40 Hz) is that they are mainly due to the mechanical effects of breathing on blood pressure (Pagani et al., 1986) but some authors have also shown a link with the endothelial derived nitric oxide (Stauss, 2007). The renin-angiotensin system and shear stress-induced release nitric oxide have been shown to affect very low frequencies in rats but need further investigation to be confirmed in humans (Stauss, 2007). The main indices of very short-term BP variability arise from spectral analysis of beat-to-beat systolic, diastolic or mean pressure; but there is a lack of studies using nonlinear methods such as fractal or entropy.

#### 2.1.3 Cardiac coherence

Cardiac coherence takes place in the general field of biofeedback which considers that physiological, cognitive and emotional systems are intimately interrelated through ongoing reciprocal communication (McCraty et al., 2006). Many methods have been developed, but globally they all try to measure the synchrony between psychophysiological interconnected systems. In this context, cardiac coherence evaluates the stability of heart rhythm in the low frequencies. Thus the cardiac coherence index will be represented by the presence of a peak in these regions visible on the spectral analysis of RR variability (Shaffer et al., 2014). From the power spectral density of the RR, the cardiac coherence ratio is defined as Peak power/[Total power−Peak power]; where Peak power is the integral in a window 0.03 Hz wide, centered on the maximum peak identified in the 0.04–0.26 Hz range; and Total power is the integral between 0.0033 and 0.40 Hz (McCraty et al., 2006).

Subjects can enhance their cardiac coherence capacity during training sessions composed of paced respiration around 0.1 Hz. In the autonomic nervous system, such training induces a resetting of the baroreceptor sensitivity, an increase in vagal afferent traffic and a reduction in sympathetic outflow (McCraty et al., 2006).

#### 2.1.4 Main fields of interest

Due to its simplicity to deploy and interpret, HRV became a gold standard to evaluate autonomic nervous system activity. Thus it is commonly used for research, clinical as well as leisure purposes. Indeed, virtually all research protocols concerning the autonomic nervous system use HRV. As some studies have demonstrated its ability to predict fatal and nonfatal events, the method is also used in clinics, especially for cardiac and diabetic populations to follow at-risk patients. Also, amateur to professional sportspersons are now accustomed to employ HRV indices to manage their training loads and to prevent overtraining, mainly through the use of HR monitor watches.

Very short-term BP variability is less popular, mainly because of the cost of the material needed to record continuous BP waveform. However, these measures have been applied successfully to cardiovascular disease, such as hypertension, heart failure and stroke; particularly to estimate sympathetic modulation of vascular tone and impaired cerebrovascular myogenic function that are predictors of stroke (Smeda et al., 1999; Kiviniemi et al., 2014; Grilletti et al., 2018; Javorka et al., 2021). As a matter of fact in these populations, very short-term BP variability also constitutes a morbidity and mortality predictor (Lucreziotti et al., 2000).

Cardiac coherence is used as an alternative treatment for certain chronic diseases, stress or pain management (Shaffer et al., 2014; Fournié et al., 2021).

### 2.2 Cardiac baroreflex

The baroreflex is the main neuronal short-to long-term regulatory system of the circulatory system (Benarroch, 2008). It adapts permanently BP, HR and blood volume (Kaufmann et al., 2020). The baroreceptors situated in the carotid sinus and the aortic arch are stimulated when arterial pressure increases, leading to bradycardia, and decrease in cardiac contractility, vascular tone and venous return. Conversely, a decrease in BP inhibits baroreceptors, generating tachycardia, and an increase in cardiac contractility, vascular resistance and venous return (Reyes Del Paso et al., 2017). Then the methods for estimating BRS are based on the analysis of HR decreases resulting from a fall in BP and HR increases resulting from a rise in BP (Pinna et al., 2017). A large response in HR resulting from a modification in BP identifies a great sensitivity of the baroreflex, since a low response indicates a blunted baroreflex. With the *CVRanalysis* software, we propose three noninvasive well known methods to calculate BRS: the sequence, the transfer function and the phase-rectified averaging methods.

#### 2.2.1 Sequence method

The sequence method aims to detect spontaneous sequences of cardiovascular changes (Parati et al., 1988; Parlow et al., 1995) i.e., consecutive increase of BP resulting in an increase in RR (positive sequence) or a consecutive decrease in BP, followed by a decrease in RR (negative sequence) (Reyes Del Paso et al., 2017). For each detected sequence, a regression line between SBP and RR is plotted and permits to calculate the slope of the curve. Then BRS is calculated as the average of all individual slopes expressed in ms/mmHg. The positive and negative BRSs can be expressed separately, and the effectiveness of the baroreflex can be calculated as the proportion in which the baroreflex is able to induce changes in response to the oscillation in SBP (Di Rienzo et al., 2001).

Some parameters can be set for the detection of sequences and the calculation of slopes: the minimum and maximum number of beat in a sequence (generally 3–6 beats), the lag between SBP and RR response (usually set to 0 to pick up the fast vagal arm of the cardiac baroreflex), the correlation coefficient of the regression line to validate a sequence (usually >0.85), and the threshold values for SBP and R-R interval changes in a sequence (typically, 1 mmHg and 1 ms, respectively) and the maximum successive R-R variations (commonly 20%).

#### 2.2.2 Transfer function

BRS can be calculated by estimating the transfer function between beat-to-beat RR and SBP series (Robbe et al., 1987; Maestri et al., 1998; Pinna and Maestri, 2001; Pinna et al., 2002). The modulus gives the gain between SBP and RR (thus corresponding to BRS in ms/mmHg), and the argument provides the phase shift between the two signals (in radian but more generally expressed as a delay in seconds). In practice, these indices are calculated in the LF and the HF frequencies as their average in the two bandwidths (Clayton et al., 1995; Pinna and Maestri, 2002) and validated when the coherence function between RR and SBP is superior to 0.50 in the corresponding LF or HF bandwidths. Also, the delays between SBP and RR are considered as valid only if they suit with physiological timing of a working baroreflex, i.e., between 0.24 and 4 s (Keyl et al., 2001; Milan-Mattos et al., 2018).

#### 2.2.3 Phase-rectified signal averaging

The phase-rectified signal averaging method (PRSA) was introduced to overcome some weakness in the robustness of the above methods against noise and nonstationnarities present in both RR and SBP signals (Bauer et al., 2010).

The method consists of finding each SBP value that is higher than the previous one and to mark them as anchors. Then all the RR sequences centered at these anchors are aligned and averaged, thus constituting the overall HR response to SBP rises. The averaged signal allows to consider only the coupled RR–SBP signals and to eliminate noncoupled oscillations such as noise and artefacts. Finally, BRS is expressed as the magnitude of RR variation solely (in ms) (Bauer et al., 2010) or normalized by the average SBP increase that triggered the RR changes (in ms/mmHg) (Muller et al., 2012).

#### 2.2.4 Main fields of interest

Conversely to HRV which provides a global status of the autonomic nervous system of a subject, BRS indices focus on the short-term neuronal regulation system of BP. Due to this specificity, such indices are preferably used on cardiac and hypertensive populations (Maestri et al., 1998; Pinna et al., 2015), in which it has been demonstrated to be a predictor of cardiovascular and all-cause morbidity and mortality (La Rovere et al., 1998; La Rovere et al., 2009; Bauer et al., 2010).

### 2.3 Time-frequency analysis of RR, blood pressure and baroreflex sensitivity

All indices described above are defined for steady state recordings and then provide physiological indices but cannot demonstrate transient variations. To overstep this limitation, one can use time-frequency analysis.

A popular tool to achieve such a possibility is the wavelet transform. First, a set of wavelets of different width (namely, different levels) is constructed from a referenced mother wavelet. Then the temporal signal is decomposed at different levels with each wavelet. Each level represents a particular frequency range from which the standard VLF, LF and HF variability indices are derived. Such analysis permits to follow the time-frequency of HRV as well as BP variability. Also, the sliding ratio between HRV and SBP variability in the LF and HF bandwidths provides the evolution of BRS along time. For a complete description of the method utilized in the software, readers are invited to refer to previous articles (Pichot et al., 1999; Wiklund et al., 2002).

However, we must be careful not to think that there are no longer any precautions to be taken when interpreting the results once there is no longer any constraint on the stationarity of the signal. The user still need to be vigilant about the mean heart rate and respiratory rate values. A significant increase in heart rate during the period to be analysed, following exercise, for example, will mean that you are in an operating zone for which the cardiac variability indices are no longer valid (Rimoldi et al., 1992). Also, as with Fourier analysis, if the respiratory rate falls outside the defined HF bandwidth, the interpretation of HF and certainly LF and LF/HF will no longer be valid.

The time-frequency analysis has been used to follow autonomic nervous system activity or BRS during tilt test (Jasson et al., 1997), pharmacologically induced changes in BP (Wiklund et al., 2002), pharmacologic blockade (Pichot et al., 1999; Pichot et al., 2001), exercise (Tiinanen et al., 2009), experimental pain (Chouchou et al., 2011), generalized interictal EEG discharge (Sforza et al., 2014) or sleep apnea (Chouchou et al., 2014), for example.

### 2.4 Granger causality

Causality is a generic term meaning cause-effect relationships between systems, subsystems, processes, or phenomena. For research in cardiovascular homeostasis, we study processes such as physiological signals focusing on RR, BP and RE. Causality analysis between RR, BP and RE can reveal the mechanisms governing RR ↔ BP ↔ RE dynamical interactions. The causality definition given by Granger in the field of multivariate stochastic processes provided a framework for estimating causality in time series (Granger, 1980). Given a set of M signals, Ω , describing the behavior of a system, the time series $X_{j}\left(t\right)$ causes $X_{i}\left(t\right)$ in Ω if the inclusion of past observations of $X_{j}\left(t\right)$ reduces the prediction error of $X_{i}\left(t\right)$ . In the literature, Granger causality can be described according to time series and frequency domain approaches.

#### 2.4.1 Time domain

Assessing causality from the Granger approach is the most popular, including the ‘MVGC multivariate Granger causality’ toolbox (Barnett and Seth, 2014). Granger causality or classical Granger causality (cGC) is a popular tool for the user for assessing the presence of directional interactions between two time series of a multivariate data set (Wiener, 1956; Granger, 1969). However, cGC only includes the time-lagged effects between processes. In respiratory and cardiovascular physiology, significant instantaneous effects are present. Subsequently, cGC may lead to an incomplete description of the real phenomenon between processes. As a possible solution, the utilization of an extended model accounting for both instantaneous and lagged effects has been proposed. This modelling is named extended Granger causality (eGC). In a formal point of view, the cGC( i→j ) is the logarithmic measure of the ratio between the variance of the residuals ${\tilde{U}}_{j}$ of the restricted regression and the variance of the residuals $U_{j}$ of the unrestricted regression.

Accordingly, the eGC is the logarithmic measure of the ratio between the variance of the residuals ${\tilde{W}}_{j}$ of the restricted regression including the zero-lag effects matrix $B\left(0\right)$ related to instantaneous effects (Faes, 2014; Schiatti et al., 2015) and the variance of the residuals $W_{j}$ of the unrestricted regression also including $B\left(0\right)$ .

#### 2.4.2 Frequency domain

Notions of causality are commonly formalized in the context of a multivariate autoregressive (MVAR) representation of time series in order to allow time and frequency domain pictures (Faes et al., 2013a). Several frequency domain measures of causality have been introduced. Actually, measures to quantify causality in the frequency domain have been proposed from strictly causal MVAR representation: directed transfer function (DTF) (Kamiński and Blinowska, 1991), directed coherence (DC) (Baccala et al., 1998) and partial directed coherence (PDC) (Baccala and Sameshima, 2001). Faes and Nollo have extended DC and PDC measures to extended causal MVAR representation to provide extended directed coherence (eDC) and extended partial directed coherence (ePDC) (Faes and Nollo, 2010). A synthesis of all measures of causality and coupling has been proposed by Faes and Nollo (Faes and Nollo, 2011). Table 5 shows the frequency domain measures of causality presented in this paper.

**TABLE 5:** Frequency domain measures of causality between two processes $X_{i}\left(t\right)$ and $X_{j}\left(t\right)$ .

Direct		Strictly causal MVAR representation	Extended MVAR representation
1) Direct causality	$X_{i}\left(t\right)\to X_{j}\left(t\right)$	PDC: $\pi_{ij}\left(f\right)$	ePDC: $\chi_{ij}\left(f\right)$
2) Extended direct causality	$X_{i}\left(t\right)\dot{\to }X_{j}\left(t\right)$		
Direct + indirect			
1) Causality	$X_{i}\left(t\right)\Rightarrow X_{j}\left(t\right)$	DC: $\gamma_{ij}\left(f\right)$	eDC: $\xi_{ij}\left(f\right)$
2) Extended causality	$X_{i}\left(t\right)\dot{\Rightarrow }X_{j}\left(t\right)$		

#### 2.4.3 Physiological interpretation

Pharmacological and basic physiological manoeuver studies have given some information on the underlying mechanisms supported by each RR, SBP and RE causal relationships (Faes et al., 2011; Faes et al., 2013b; Faes and Nollo, 2011; Porta et al., 2013b; Porta et al., 2014; Porta et al., 2015). Then it has been demonstrated that the causal link between SBP and RR is due to baroreflex involvement, as the opposite link between RR and SBP has been hypothesized to be due to Starling law and arterial Windkessel effect (Porta et al., 2011). The coupling between respiratory centers and the cardiac vagal center in brainstem, the activation of cardiopulmonary reflexes and direct mechanical pulmonary stimulation of the sinus node tissue through inspiratory inflations, constitute the physiological mechanisms of the relationship between RR and RE (Saul et al., 1991; Eckberg, 2003). The causal relationship from RE to SBP is due to variations of stroke volume resulting from mechanical effects on intrathoracic pressure and venous return (Toska and Eriksen, 1993; Caiani et al., 2000). Finally, no physiological mechanisms were show for the causality link between SBP to RE (Porta et al., 2013b).

#### 2.4.4 Application

The main studies using Granger causality analyses concern the physiological validation of the methods and some mathematical improvements, such as the extended version of the Granger causality which is an adaptation of the method to physiological constraints (Faes and Nollo, 2010; Schiatti et al., 2015). Some authors tested successfully the causal method on anesthesia (Bassani et al., 2012; Porta et al., 2013a), syncope (Faes et al., 2013b; Faes et al., 2015; Schiatti et al., 2015), pre-eclampsia (Riedl et al., 2010), or fibromyalgia patients (Zamunér et al., 2017) but there remains a lack of major clinical studies using these indices as prognostic values.

## 3 Software description

The software allows the analysis of RR, SBP, MBP, DBP and RE series on short duration recordings, lasting typically several minutes to hours. The software is not optimized for the analysis of data arising from 24-h ambulatory recorders and users are invited to refer to other software for such treatments (Pichot et al., 2016).

To carry out data analyses, users are invited to follow three steps: 1) data importation and beats detection, 2) beats corrections and formatting, and 3) data analyses. For each step, an undo process is possible, allowing the user to easily find again the initial preprocessed data.

The software has been written to give nonprogrammer users access to a wide range of analysis tools. The user is guided and default settings are proposed. However, incorrect use of *CVRanalysis* can lead to erroneous results. For this reason, users of the software must master all the steps leading to the results of an analysis. Before validating a result, users should check the following:

- the state of the subject in which the chosen analysis was carried out (steady state or transition period, for example)
- the shape and values of the raw signals are correct, particularly with regard to the presence of R peaks and BP values
- artefacts have been corrected correctly
- the number of artefact corrections is not too large; generally less than 2% of the analyzed beats.
- the analysis method is appropriate for the purpose and can be used on the data,
- the parameters of the analysis methods used have been correctly chosen, particularly when the subjects analysed are young, for example.

### 3.1 Main window

**FIGURE 1:**
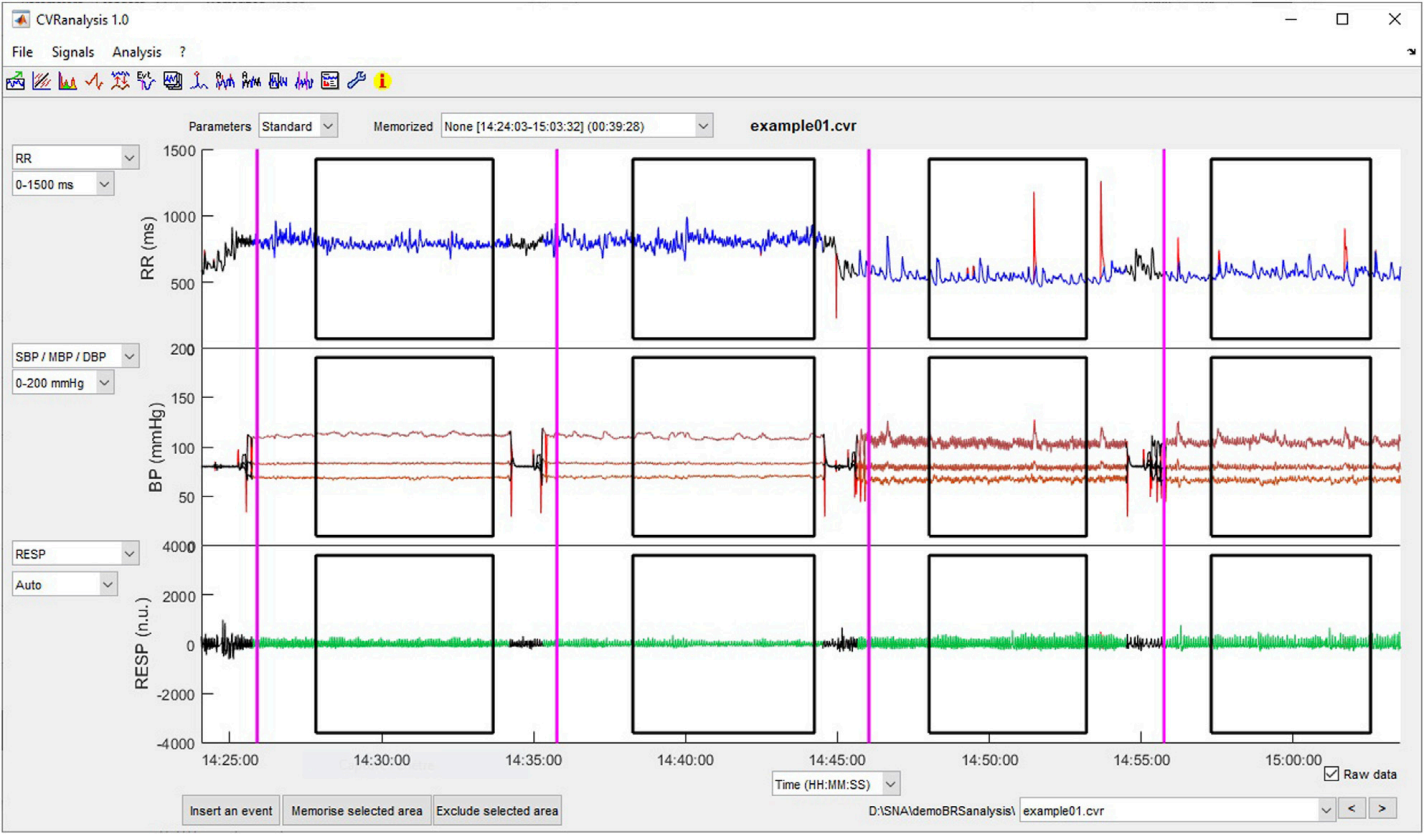
Main window of *CVRanalysis* software.

The main figure displays the signals and gives access to the different functions of the software by using the menus or pushbuttons or the toolbar shortcuts (Figure 1).

Three axes permit to plot the raw or beat-to-beat data, one signal per axis. The plotted data are selected from the popup menus on the left part of the axes. The user can choose the x-axis in seconds or in beats, using the popup menu below the third axis. Also, the scale of the y-axis can be automatic or selected manually from a list of preset values. The raw values of the RR, BP and RE signals are plotted in red as the corrected ones; those will be used for the analyses are plotted in blue (RR), brown (BP) and green (RE). Excluded parts of the signals are in black color. The vertical purple lines represent the events and the black squares surround the areas of interest, both entered by the user (see description below). Raw uncorrected beat-to-beat data, selected areas, and event can be individually displayed/undisplayed by checking/unchecking them in the “Signals” menu. On each axis, it is possible to zoom in on the data by pressing the left mouse button while selecting the zone to be magnified. This procedure can be repeated to refine the view, and a left mouse double-click returns the view to the full data plot. When zooming in on a plot, the x-axis of the three plots are automatically set to the same values in order to always have a synchronous view of the data.

A popup menu situated on the upper right part of the window allows the user to have a quick access to all .cvr files of the current directory. Also, a back (<) and a forward (>) pushbutton permit to shift to the previous or next file, respectively.

### 3.2 Preferences setting and tutorial

A Preferences window allows the user to modify and set the analyses parameters and a Tutorial is accessible from the main window.

For each method, the software offers default parameter settings. The values have been chosen either because they have been standardized or because they are the most commonly used. Of course, the user has the option of modifying certain settings. Three configurations for which the parameters have been set are “Standard” (usual settings for adults), “Children,” and “Newborn.”

The default and modifiable parameter values are summarized in Table 6. We have chosen to leave some parameters unmodifiable, generally because they are always used with the same values in published studies; this can be seen as a limitation of the software.

**TABLE 6:** List of methods parameters for the analyses used in the software. Some parameters can be modified by the user as others are fixed.

Power spectral density		
Type	**[Non-parametric FFT]**, parametric AR	Editable
Detrend	**[yes]**, no	Editable
Resampling frequency (Hz)	None, 1, **[2]**, 4, 8 [4]* [8]**	Editable
Bandwidth HRV, baroreflex		
VLF (Hz)	**[0–0.04]** [0–0.02]**	Editable
LF (Hz)	**[0.04–0.15]** [0.02–0.20]**	Editable
HF (Hz)	**[0.15–0.40]** [0.15–1.40]* [0.15–2.00]**	Editable
Bandwidth BPV		
VLF (Hz)	**[0.02–0.07]**	Editable
LF (Hz)	**[0.07–0.15]**	Editable
HF (Hz)	**[0.15–0.40]** [0.15–1.40]* [0.15–2.00]**	Editable
Number points/Hz	**[256]**	Fixed
Welch periodogram		
Window	**[Hanning]**, Hamming	Editable
Window width	[**1024**]	Fixed
Window overlap (%)	0, 25, **[50]**	Editable
**Entropy**		
Approximate entropy		
Embeded dimension m	**[2]**	Editable
Tolerance r (*SD)	**[0.2]**	Editable
Sample entropy		
Embeded dimension m	**[2]**	Editable
Tolerance r (*SD)	**[0.2]**	Editable
**Symbolic dynamics**		
ξ	**[6]**	Fixed
L	**[3]**	Fixed
**Largest Lyapunov exponent**		
m	1, 2, **[3]**, 4, … 20	Editable
t	**[20]**	Fixed
T	**[3]**	Fixed
Smax	**[0.3]**	Fixed
Smin	**[0.001]**	Fixed
thmax	**[30]**	Fixed
**Fractal**		
DFA		
n1	**[4–11]** [4–39]* [4–39]**	Editable
n2	**[12–100]** [40–100]* [40–100]**	Editable
Higuchi		
Kmax	**[6]**	Fixed
**Baroreflex**		
Sequence method		
nb minimum beats/ramp	**[3]**, 4, 5, … 10	Editable
nb maximum beats/ramp	3, 4, 5, **[6]**, … 10	Editable
Lag	**[0]**, 1, 2	Editable
Minimum BP variation (mmHg)	**[yes]**, no [1], 2, 3	Editable
Minimum RR variation (ms)	**[yes]**, no [1], 2, 3, 4, 5	Editable
Maximum RR variation (%)	**[yes]**, no 10, 15, [20], 25, … 50	Editable
Valid ramp for r >	**[yes]**, no 0.50, 0.55, … [0.85], 0.90, 0.95	Editable
Outliers selection	None, **[Auto]**, Manual	Editable
Transfert function		
Valid for coherence >	**[yes]**, no [0.50], 0.60, 0.70, 0.80, 0.90	Editable
Phase-Rectified Signal Averaging		
Segments length (2L)	**[30]**, 40, 50, … 100	Editable
**Granger causality**		
Number lags max	1, 2, … **[20]**, … 100	Editable
Causal direction (extended)	“General entropy-based method (any var distribution)”	Editable
	**[“First-order approximation of LR by tanh (sparse var)”]**	
	“Basic skewness measure (skewed var)”	
	“New skewness-based measure (robust to outliers)”	
	“Dodge-Rousson measure (skewed var)”	
Causal direction (classic)	**[Gaussian]**	Fixed

In bold between square brackets: default values for “standard” mode; *default values for “baby” mode; **default values for “newborn” mode.

### 3.3 Data importation formats

Data can be imported as raw signals (ECG, continuous BP and respiratory wave forms) or as beat-to-beat signals (RR, SBP, MBP, DBP, RE). At least ECG and continuous BP or beat-to-beat RR and SBP are necessary to perform the analyses. Diastolic, mean BP and respiratory signals are optional and can be omitted.

**FIGURE 2:**
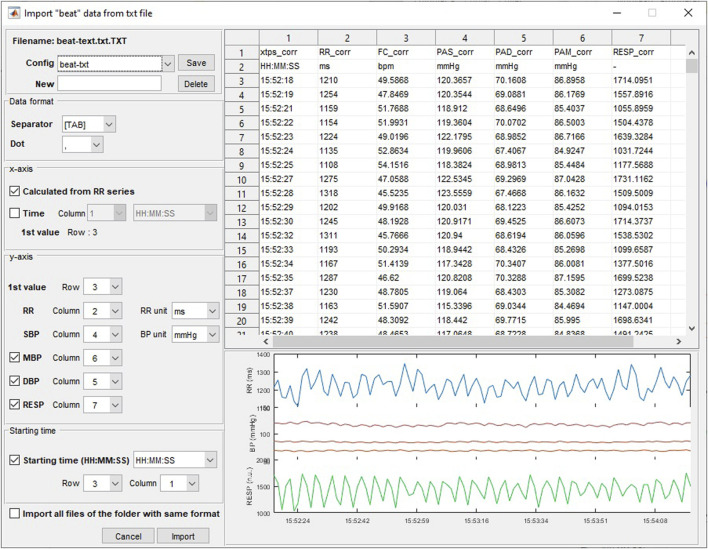
Importation of beat-to-beat data from a file in.txt format.

The available formats are EDF, binary and MATLAB for raw data; and text, binary and MATLAB for beat-to-beat data. For each format, a generic import window allows the user to set the data file specifications and to previsualize the data (Figure 2). Preformatted file configurations can be set and saved by the user.

The original file data is not modified and a .cvr file dedicated to the software is automatically created when importing data. This file contains the initial data and all corrections and formatting that have been done by the user and can be opened directly from the “file/Open CVR file (*.cvr)” menu or the corresponding toolbar for further work.

### 3.4 R-peaks, systolic, mean and diastolic pressure, and respiration detection

**FIGURE 3:**
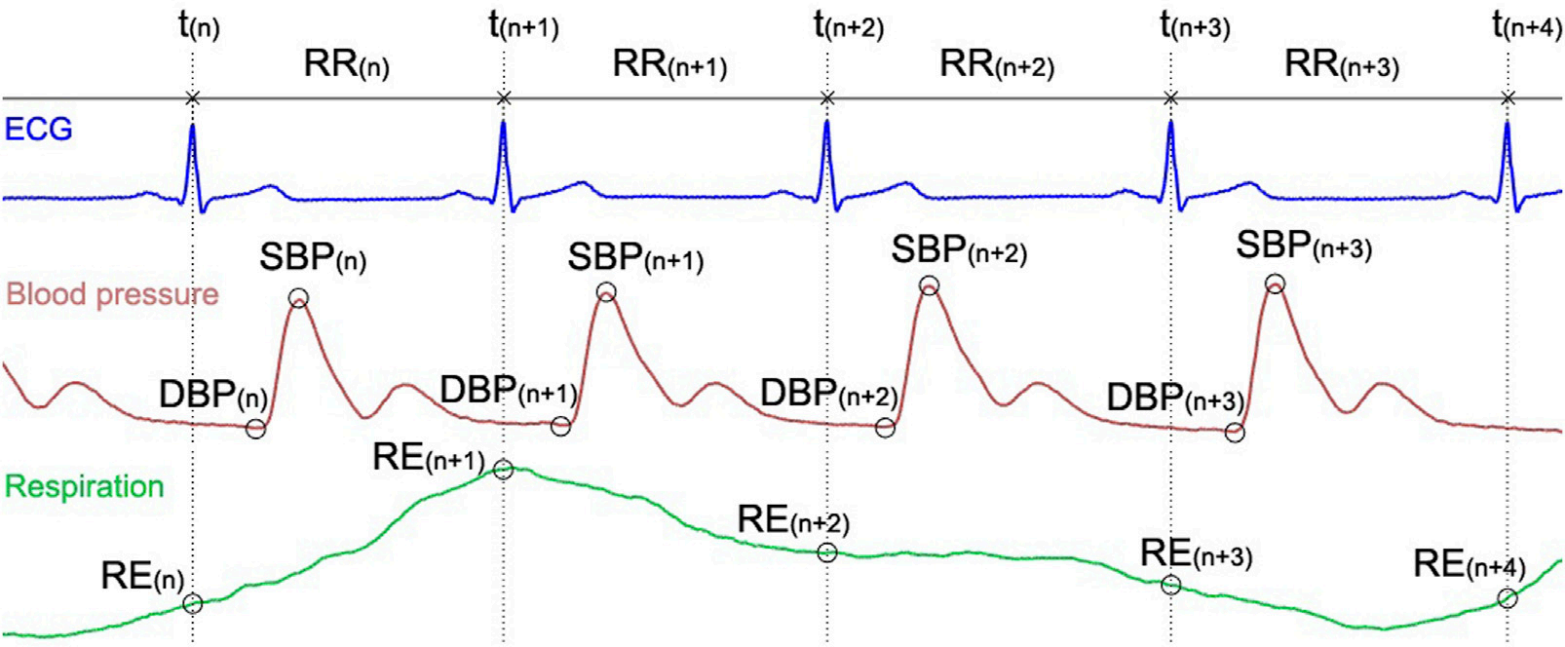
ECG, blood pressure, and respiratory signals and the location of the corresponding beat-to-beat RR, SBP, DBP and RE extracted data.

When importing raw signals, the software automatically converts them to beat-to-beat series. First, R peaks are detected from the ECG signal using a laboratory developed algorithm (Pichot et al., 2016). Then maximum and minimum BP values between RR are classified as systolic and diastolic BP, respectively (Goreham et al., 2017). The MBP value is also calculated as the mean of the blood pressure curve taken between the successive diastoles. If a respiratory signal is present, the values corresponding to the R-peak localization time are taken as the beat-to-beat value for the respiration. An example of a typical raw recording and its corresponding beat-to-beat points is presented in Figure 3.

### 3.5 Beat corrections and exclusion

The calculation realized on short duration recordings do not tolerate artefacted data that will induce erroneous analysis values and then miscellaneous physiological interpretation (Saul et al., 1988).

Indeed, ECG artefacts, missing beats, BP calibration, artefacts due to movements can be present in the recordings. In the software, we proposed three possibilities of correction:

- Automated corrections: RR and BP and respiration series are corrected automatically after selecting the Signals/Correction Auto or the corresponding toolbar. First, the erroneous beats are detected using a high and low threshold (+32.5% and −24.5%, respectively) for the relative variation of successive RR, SBP, MBP and DBP beats (Cheung, 1981). For the RE signal, the outliers are detected and replaced using a Hampel filter with the parameters set to 15 for the size of the sliding window and 10 for the standard deviation (Liu et al., 2004).
- Manual corrections of beat-to-beat values: The user selects the beat and changes its value either by entering a new value or by using spline cubic interpolation to determine it.
- Manual correction using the ECG trace: If the ECG trace is available, the user can delete/insert R-peaks by editing the ECG; after which, the new RR, SBP, MBP, DBP and RE values are updated.

These corrections make sense for recordings containing few erroneous beats, but it is obvious that they will not save very corrupted signals. The areas containing too many uncorrected beats can be excluded from an analysis; this can be done manually by selecting the areas or automatically on the entire recording by selecting the corresponding menu or toolbar. At any time, it is possible to undo the corrections and exclusion in order to get back to the preprocessed data.

### 3.6 Setting labeled area of interest

It is possible to set some labeled areas of interest which can be utilized as simple visual markers or for the automatic batch analysis procedure (see description below).

First, the user zooms in on the data to display the area to select, as described above. Then the selection of the “Signals/Memorise selected area” menu or “Save selected area” pushbutton opens a window in which the user will enter a label, refine the area limits if necessary, and confirm or cancel the selection. If the option is checked in the “Signals” menu, the new area will appear as a black square surrounding the area. All entered events can be managed (rename and delete) in a dedicated window called by selecting the “Signals/Manage areas” menu or the corresponding shortcut pushbutton.

### 3.7 Setting labeled events

The user can also insert labeled events. After selecting the “Signals/Insert an event” menu or its corresponding pushbutton, the user positions the cursor on the correct location and validates it with a left-click on the mouse. To improve precision in the cursor location, it is recommended to zoom in on the data before selecting the event position. After validation, a window automatically opens where the user will set a label and refine the event location if necessary, and finally confirms or cancels the selection. As well as for the labeled area of interest, the user can edit and manage all the events using the menu “Signal/Manage selected areas.” From this last window, it is also possible to import a list of preset events and saved in a text file.

### 3.8 Data analyses

A wide range of analyses is proposed in the “Analysis” menu or accessible directly from the toolbar shortcuts. The analyses are performed on the part of the data selected in the main window using the zoom in tool. For analysis of cardiac and BP variability, barorefelex and Granger causality, the duration selected must be between 2 min and 1 h. The lower bound corresponds to the minimum duration required to estimate very low frequency (VLF) variations. The upper limit was set empirically; it seems to us to be a reasonable maximum value during which the subjects are in a stable state. This stability is estimated by analyzing the stationarity about its mean, variance and autocovariance with a gamma value equal to 0.90 of the RR, BP and RE signals. These stationarity indices and the number of corrected beats are indicated in the analysis windows.

Each type of analysis is displayed in a dedicated window according to the parameters set in the preferences window, although some of them can be changed directly from the window. The user can export the results as a.txt file, save and print the figure. The results can be appended to an existing file to allow combining of analyses derived from different parts of the signal and/or different files. Results are then ready to use in a statistical analysis software.

#### 3.8.1 Heart rate and blood pressure variability and cardiac coherence

**FIGURE 4:**
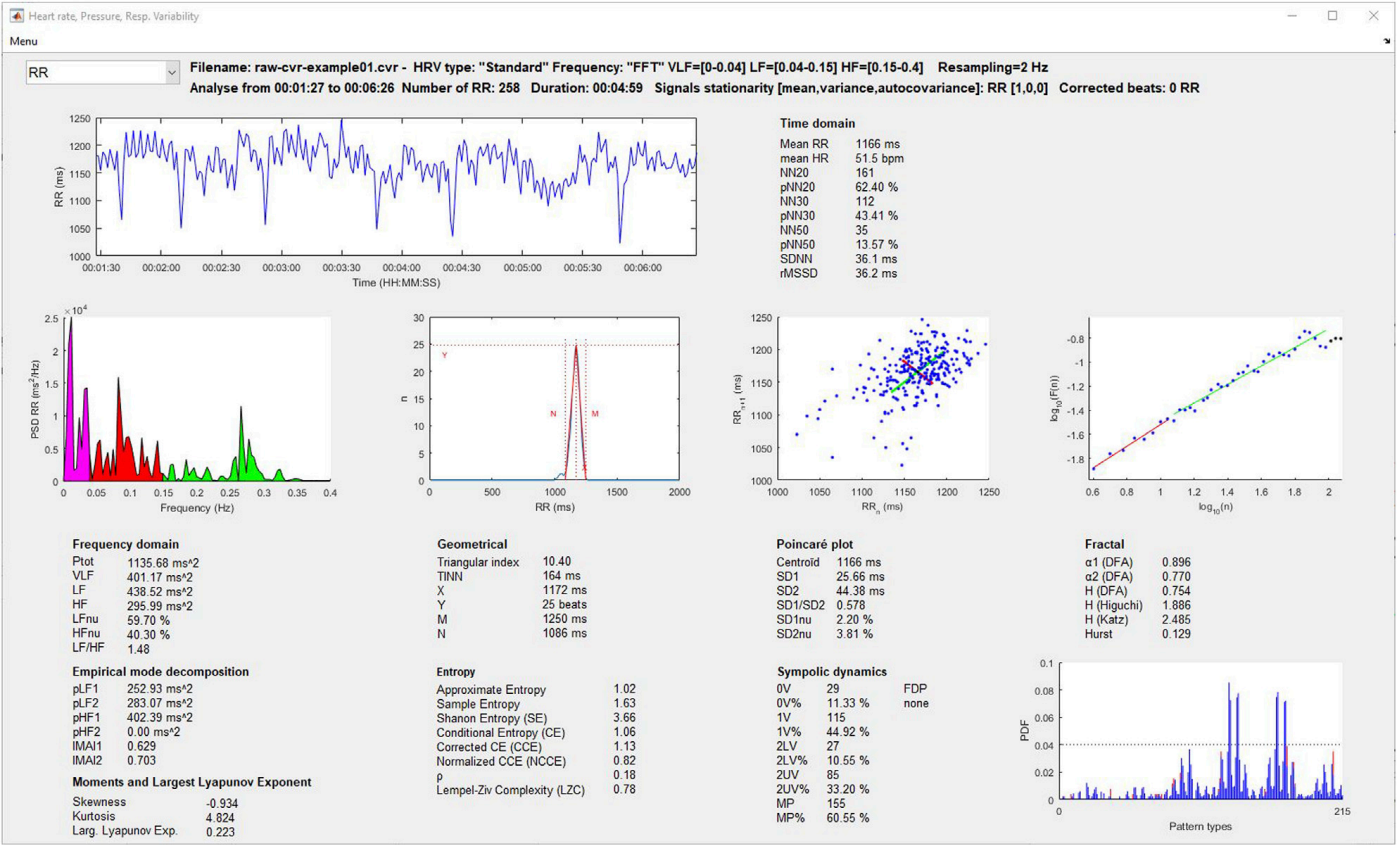
Heart rate and blood pressure variability analysis window.

**FIGURE 5:**
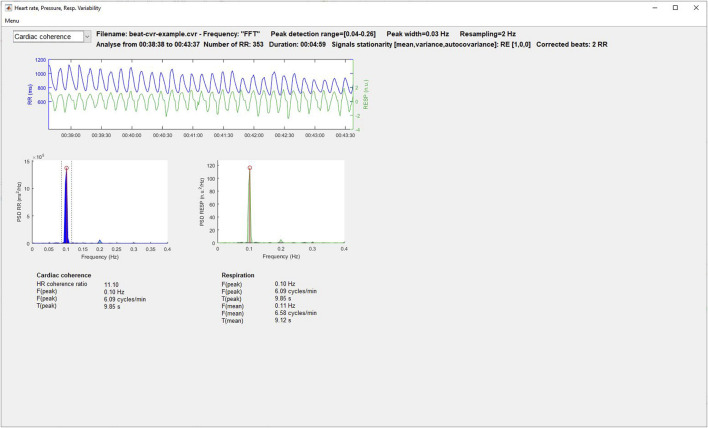
Cardiac coherence analysis window.

Time and frequency domain, geometrical, empirical mode decomposition, moments and Lyapunov exponent, entropy, Poincaré plot, symbolic dynamics, and fractal indices of variability are displayed in Figure 4. The user can select the signal to analyze (RR, SBP, MBP, and DBP) using a popup menu, after which the results are automatically updated. Some graphs of the most recognized analyses are plotted. Additionally, the user can select the cardiac coherence analysis from the same popup menu (Figure 5).

#### 3.8.2 Baroreflex indices

**FIGURE 6:**
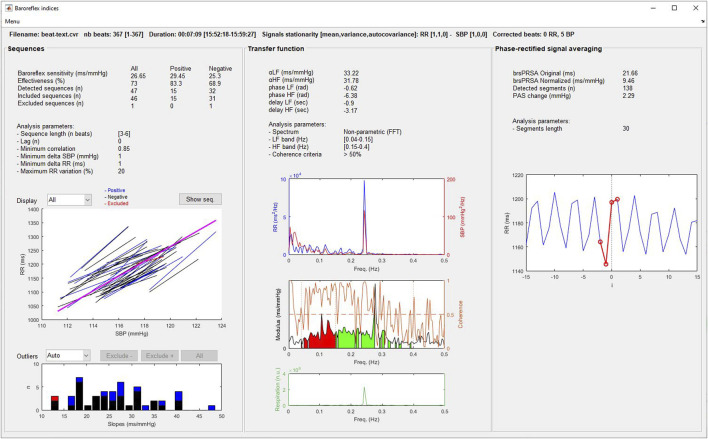
Baroreflex analysis window.

The results provided by the three methods described above (sequences, transfer function and phase-rectified signal averaging) are displayed in Figure 6. For the sequence method, the user can exclude the outlier slopes manually or automatically (exclusion of the sequences out of mean ± 3*SD). Positive, negative and excluded sequences are plotted in blue, black and red, respectively. Also, the localization of each detected sequence can be displayed on an additional window by selecting the “Show seq.” pushbutton.

#### 3.8.3 Wavelets analysis

**FIGURE 7:**
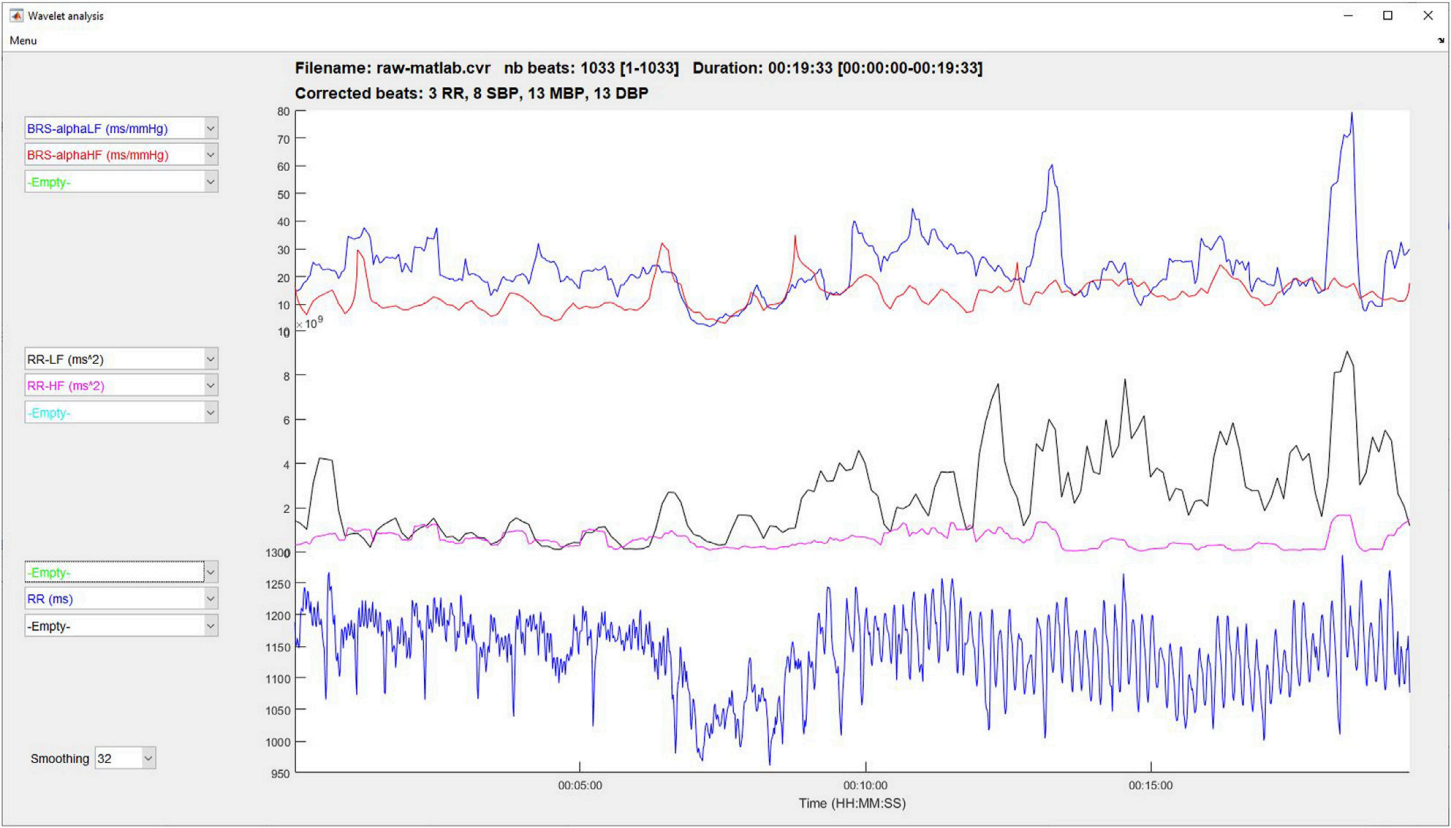
Time-frequency analysis using wavelet transform.

HRV, BPV, and baroreflex indices are calculated using wavelet transform, as described in the Signal processing section. Three graphs allow plotting changes in RR, HR, SBP, MBP, DBP and the LF, HF, LFnu, HFnu, and the LF/HF ratio for RR and BP signals along time (Figure 7). The displayed signals are selected from three popup menus on the left side of each graph. If entered, the events and selected areas are plotted and the user can zoom in on the data using a synchronized scale for the three graphs. On the bottom of this Figure, a popup allows the user to select the number of points of the sliding average performed on the signals.

#### 3.8.4 Granger causality

**FIGURE 8:**
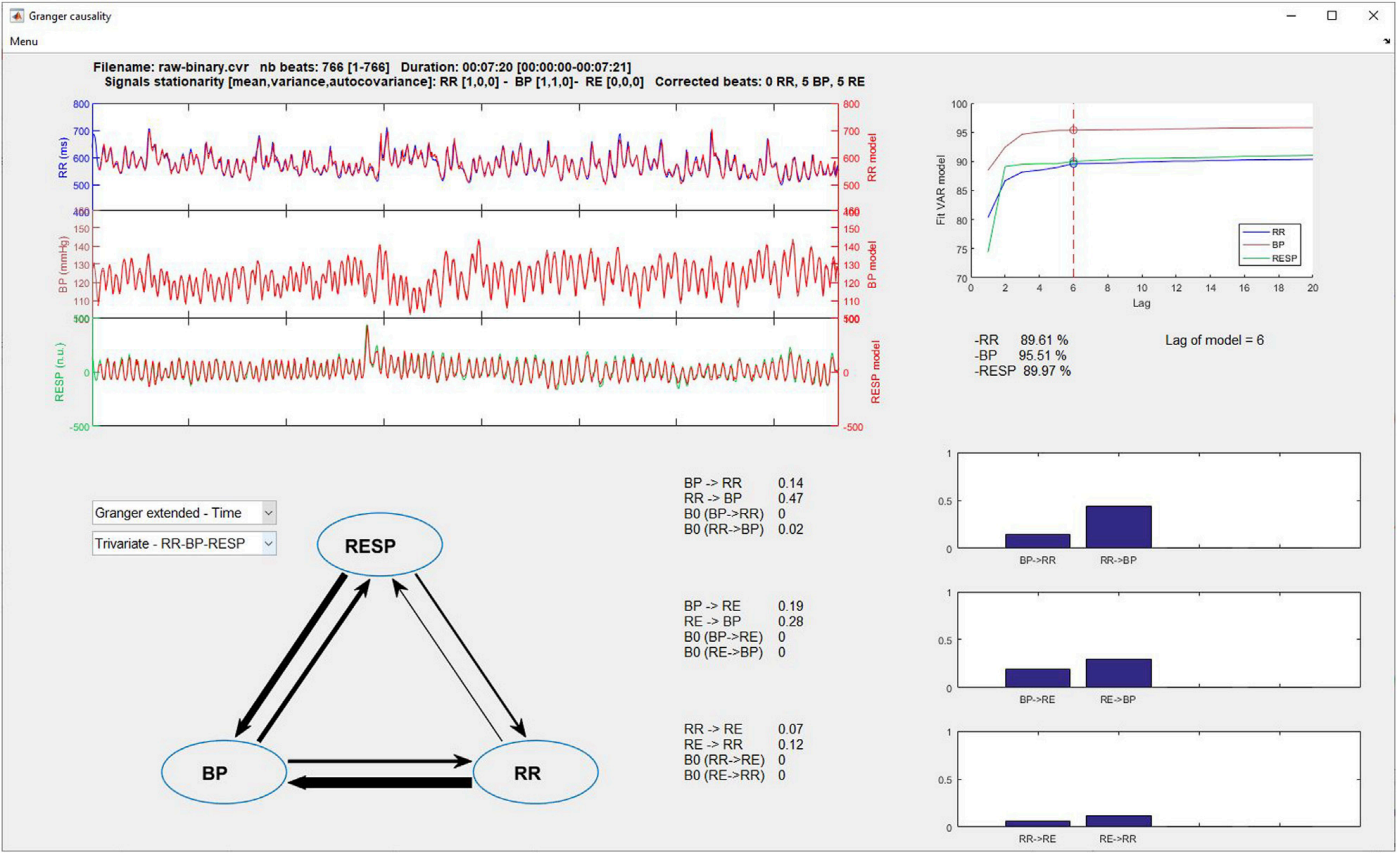
Granger time domain analysis window.

**FIGURE 9:**
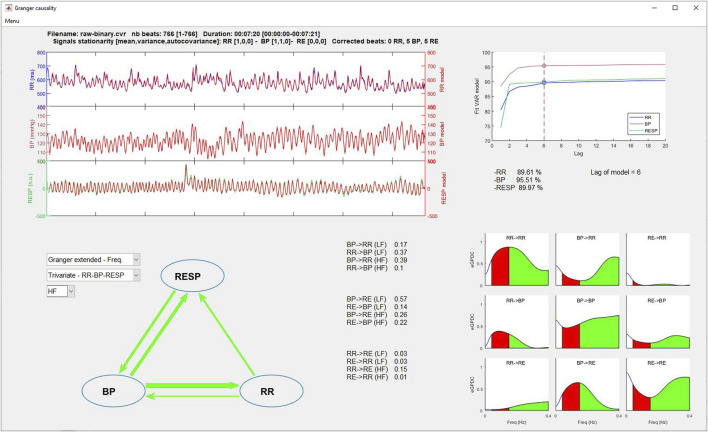
Granger frequency domain analysis window.

The upper left part of Figures 8, 9 displays the original beat-to-beat signals and their modelizations (red color), while the upper right part gives the lag and the fit model values for each of them, allowing the user to evaluate the adequacy of the model for the calculation of Granger causality indices.

On the lower left part, two popup menus permit to select the type of Granger causality analysis to display (time or frequency domain and classical or extended) and the number of signals to consider (bivariate or trivariate analysis). Results are displayed in three manners: 1) a symbolic representation of the causality indices using arrows between each signal, with the thicker the arrow, the greater the causality and vice versa; 2) the numerical values of each causality; and 3) the causality indices represented as bar graphs (time domain) or spectrums (frequency domain) for easier comparisons.

#### 3.8.5 Analysis of surrounding events

**FIGURE 10:**
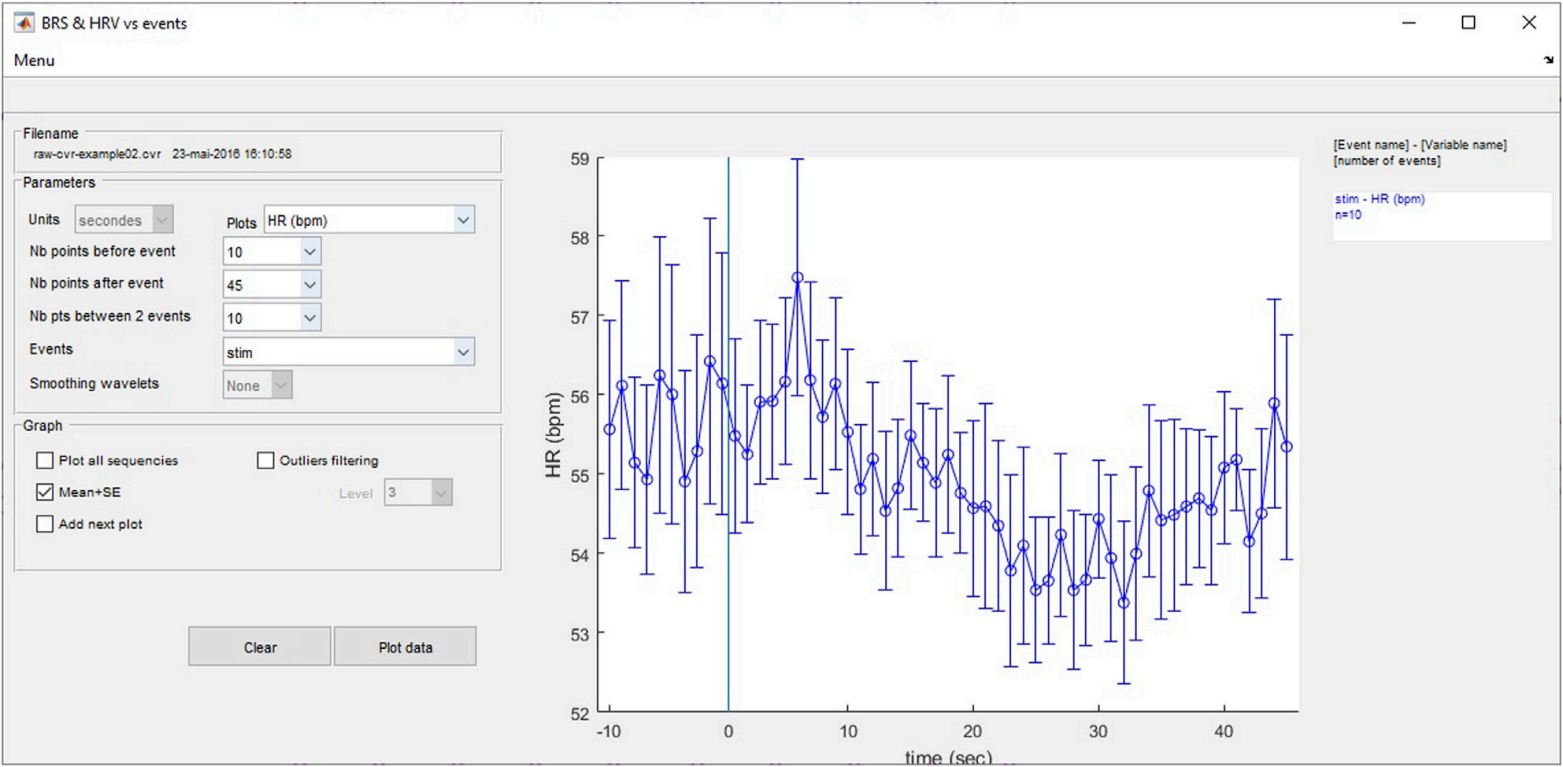
Heart rate and blood pressure variability and baroreflex analyses around user-entered events.

This analysis displays the beat-to-beat evolution before and after an event of RR, HR, SBP, MBP and the variables Ptot, VLF, LF, HF, LFnu, HFnu, LF/HF alphaLF and alphaHF calculated by the wavelet method. If several events with the same name have been entered, the program calculates the average of the changes in the chosen variable synchronized around the event. In this window, the user selects the variable to be analyzed, the event around which the analysis is to be performed, and the durations to be considered before and after the event using the appropriate popup menu (Figure 10). When the user selects the “Plot data” pushbutton, the program searches all the sequences that meet the entered criteria and plots the results. The variables are resampled at 1 Hz to allow a regular 1-s step plotting. Comparisons are possible by adding multiple analyses using different HRV, BPV and baroreflex indices and/or different events on the same graph. Results can be plotted with all sequences or as the mean ± SD, and an outlier filtering can be applied.

#### 3.8.6 Batch analyses

All analyses described above can by computed in batch mode to avoid repetitive operations and to enable the analysis of a wide number of files. Before using this tool, users have to prepare all files as follows (all steps are described above): 1) import all data to create the .cvr files; 2) make all corrections and exclusions of the beat-to-beat data; and 3) set all the areas of interest and/or events with which the batch analyses will be perform. Then after having selected the File list analysis in the ‘Analysis’ menu or by pressing the corresponding toolbar, the batch analysis window opens. First, the user selects the folder containing the .cvr files to analyze. For each analysis, the user selects the options in the windows; for the analyses, the software will use the parameters set in the preferences window. After pressing the pushbutton corresponding to the desired analysis, the software will analyze all areas and save the results in a.txt file directly importable and ready for use in statistical software environments.

## 4 Hardware specifications and system requirements

*CVRanalysis* was developed using MATLAB 2016a and compiled using MATLAB compiler 6.2. Some functions previously developed and unselfishly distributed by other programmers were used and adapted for the software: many thanks to Faes (2017) and Schiatti et al. (2015) (Granger causality), Wenye G (detrended fluctuation analysis), Alvarez JM (entropy, Katz and Higuchi indices), Rilling G and Flandrin P (empirical mode decomposition), Wolf A (largest Lyapunov exponent), and Zhivomirov H (stationarity test).

It is not necessary to have MATLAB installed on the computer since MATLAB Runtime v9.0.1 is automatically installed during the software setup process. *HRVanalysis* works with Windows 64-bit operating systems.

As some calculations are time consuming, it is recommended to have at least 4 GB of RAM on the computer. Also, a minimal screen size of 1280 × 768 allows to display all windows without problem. The user has to make sure that there is no magnifying factor for the characters and applications set in the graphic parameters of the Windows system.

## 5 Tutorial and sample runs

A tutorial containing a detailed description of the software is directly accessible from the main menu, and some sample runs to help users learning and testing the software’s functioning are included with the software package.

## 6 Availability, licensing and installation procedure

*CVRanalysis* is available freely for noncommercial use only (Freemium license, 8 February 2022, under the number IDDN.FR.001.060017.000.S.P.2022.000.31230).

The software can be downloaded from the dedicated webpages of anslabtools.univ-st-etienne.fr. Before downloading, a registration is required so that users may be kept informed of free software updates.

*CVRanalysis* is installed by launching the installer program and the user will be invited to validate the license when running the software for the first time.

We will be pleased to receive suggestions, comments or bug reports concerning the program at the following e-mail address: ANSLabTools@univ-st-etienne.fr. Also, users are asked to cite the present article and the software download pages when they used it for the analysis of published data.

## 7 Conclusion

*CVRanalysis* has been used and improved for over 20 years by the SNA-EPIS, and now SAINBIOSE laboratories, Saint-Etienne; thus suitable for research laboratory requirements. The software is perfectly adapted for numerous fields such as death and health prediction, cardiac and respiratory rehabilitation, training and overtraining, large cohort follow-ups, diabetes, and children’s autonomic status, anesthesia, pain, or neurophysiological studies.

The main strength of *CVRanalysis* is its wide scope of application and intuitive human-machine graphical interface. The software does not require MATLAB software and there is no need for the user to have programming skills. In addition to standard baroreflex and variability analyses, the software allows time-frequency analysis using wavelet transform, time and frequency domain Granger causality analyses, and analysis of autonomic nervous system and baroreflex status surrounding scored events and on preselected labeled areas. Each analysis can be exported as a figure or text file and directly usable in a statistical software. Moreover, the batch signal processing tool facilitates large cohort analysis.

## Data Availability

The raw data supporting the conclusion of this article will be made available by the authors, without undue reservation.
